# Data source mapping: an essential step for health inequality monitoring

**DOI:** 10.1080/16549716.2018.1456743

**Published:** 2018-05-16

**Authors:** Ahmad Reza Hosseinpoor, Devaki Nambiar, Suparmi, Nunik Kusumawardani

**Affiliations:** a World Health Organization, Geneva, Switzerland; b George Institute for Global Health, Delhi, India; c Indonesia Agency for Health Research and Development, Jakarta, Indonesia

**Keywords:** Monitoring Health Inequality in Indonesia, Data source mapping, health inequality monitoring, Indonesia

## Abstract

The task of health inequality monitoring is not possible without the availability of appropriate and high-quality data at various levels. Data source mapping – a process by which data sources are systematically enlisted, their properties detailed and each source appraised for the purposes of monitoring – is an essential initial step for health inequality monitoring. We outline a simple process along with a template for data source mapping and its application in Indonesia, concluding with the lessons learned from this process, in terms of both challenges as well as the opportunities and advantages arising from the use of equity-related data from the Indonesian health information system.

The task of health inequality monitoring, the importance of which is underscored in this special issue [1], is not possible without the availability of appropriate and high-quality data. Carrying out this task aligns with the framework and requirements of the health Sustainable Development Goal, and also meets the requirement of Target 18.17 which calls for the availability of disaggregated data of high quality, that is routinely available and reliable [2].

**Sheet 1.** Types of national level data sources.

Type	Name	Year
Census	Population census	1961, 1971, 1980, 1990, 2000, 2010
Institution-based	Indonesia health profile (report including health centres and hospitals)	Annually
Survey	RISKESDAS (Basic Health Research)	2007, 2010, 2013
Survey	RIFASKES (Health Facility Survey)	2011
Survey	SIRKESNAS (National Health Indicators Survey)	2016
Survey	TB Prevalence Survey	2004, 2014
Survey	SUSENAS (National Socioeconomic Survey)	1979, 1980, 1981, 1984, 1989, then annually
Survey	SUPAS (Intercensal Survey)	1995, 2005, 2015
Survey	GATS (Global Adult Tobacco Survey)	2011
Survey	GYTS (Global Youth Tobacco Survey)	2006, 2009, 2014
Survey	IDHS (Indonesia Demographic and Health Survey)	1987, 1991, 1994, 1997, 2003, 2007, 2012
Survey	PODES (Village Potential Survey)	1983, 1986, 1990, 1993, 1996, 2000, 2003, 2005, 2008, 2011, 2014
Survey	Global School Based Health Survey	2015
Vital registration	SRS (Sample Registration System)	2014, 2015

Source: Indonesia Agency for Health Research and Development, Authors, based on [5–20]

**Sheet 2.** Data sources listed and mapped by dimensions of inequality.

Unique data source number	Unique data source name	Dimension of inequality						
		Income/expenditure/consumption/asset index	Education	Occupation	Sex	Urban/rural	Province/region	Race/ethnicity
1	RISKESDAS (Basic Health Research) 2007 [5]	√	√	√	√	√	√	×
2	RISKESDAS (Basic Health Research) 2010 [6]	√	√	√	√	√	√	×
3	RISKESDAS (Basic Health Research) 2013 [7]	√	√	√	√	√	√	×
4	RIFASKES (Health Facility Survey) 2011 [8]	×	×	×	×	×	√	×
5	SIRKESNAS (National Health Indicators Survey) 2016 [9]	√	√	√	√	√	√	×
6	SUSENAS (National Socioeconomic Survey) [10]	√	√	√	√	√	√	×
7	SUPAS (Intercensal Survey) 2015 [11]	√	√	√	√	√	√	×
8	GATS (Global Adult Tobacco Survey) 2011 [12]	×	√	√	√	√	√	×
9	GYTS (Global Youth Tobacco Survey) 2014 [13]	×	×	×	√	√	√	×
10	IDHS (Indonesia Demographic and Health Survey) 2012 [14]	√	√	√	√	√	√	×
11	SRS (Sample Registration System) 2016 [15]	×	√	×	√	√	×	×
12	Indonesia health profile 2015 [16]	×	×	×	×	×	√	×
13	Population census 2010 [17]	√	√	√	√	√	√	√
14	PODES (Village Potential Survey) 2011 [18]	×	×	×	×	×	√	×
15	TB Prevalence Survey 2014 [19]	×	×	×	√	√	√	×
16	Global School Based Health Survey 2015 [20]	×	√	×	√	×	√	×

Sources: Indonesia Agency for Health Research and Development, Authors, based on [5–20]

**Sheet 3.** Data sources mapped by health topic/indicators.

**Health topics**	**Unique data source number**							
**Reproductive health**								
Adolescent fertility rate	2	10						
Total fertility rate	2	10						
Contraceptive prevalence – modern methods	2	10	16					
Demand for family planning satisfied	10							
**Maternal health interventions**								
Antenatal care coverage – at least four visits	2	3	5	6	10	12		
Births attended by skilled health personnel	2	3	5	6	10	12		
Postnatal care coverage	2	3	5	6	10	12		
**Child health interventions**								
Complete basic immunization coverage	1	2	3	5	6	10	12	
Vitamin A supplementation	1	2	3	5	10	12		
Exclusive breastfeeding	1	2	3	5	6	12		
**Nutrition**								
Prevalence of stunting among under-5 children	1	2	3	5				
Prevalence of obesity among adults	1	2	3	5				
Prevalence of low birth weight	1	2	3	5				
**Infectious diseases**								
Prevalence of malaria	2	3						
Prevalence of acute respiratory infection	1	3						
Prevalence of TB	15							
**Non-communicable diseases**								
Prevalence of diabetes mellitus	1	3						
Prevalence of anaemia	1	3						
Prevalence of hypertension	1	2	3					
**Injury**								
Prevalence of falls	1	3						
Prevalence of road traffic accidents	1	3						
Prevalence of serious injury	16							
**Mental health**								
Prevalence of population with psychosis/schizophrenia	3							
Prevalence of mental emotional disorder	1	3						
**Disability**								
Prevalence of disability	1	3						
**Child mortality**								
Neonatal mortality	1	10	11	13				
Infant mortality	1	7	10	11	13			
Under-5 mortality	1	7	10	11	13			
**Maternal mortality**								
Maternal mortality ratio	7	10	11	13				
**Healthy/unhealthy behaviour**								
Prevalence of current smoking	1	3	6	8	9	16		
Prevalence of alcohol consumption	1	3	8	9	16			
Prevalence of physical inactivity	1	3	16					
Prevalence of low fruit/vegetable consumption	1	3	16					
**Environmental health**								
Proportion of household using improved drinking water	1	2	3	6	13	14		
Proportion of household using improved sanitation	1	2	3	6	13	14		
Proportion of household using pesticide	1	2	3	6	13	14		
**Health insurance**								
Proportion of population with national health insurance	3	6						
Proportion of population with province/district health insurance	3	6						
Proportion of population with private health insurance	3	6						
**Health care access**								
Average travel time to health centre	1	3	14					
Average transportation cost to health centre	1	3	14					
Inpatient utilization rate	1	3	6					
Outpatient utilization rate	1	3	6					
**Health facility**								
Number of hospital by province	4	12	14					
Health centre density (per 300,000 population)	4	12						
Bed occupancy rate in public hospital	12							
**Health financing**								
Average out-of-pocket health expenditure	6							
Health expenditure per capita	6							
**Human resource for health**								
Number of doctors, midwives, nurses, nutritionists, sanitarians, health promotion staff in hospitals and in health centres	4	12						
Density of doctors, midwives, nurses, nutritionists, sanitarians, health promotion staff (per 10,000 population)	4	12						

Note: the unique data source number derives from Sheet 2.

Sources: Indonesia Agency for Health Research and Development, Authors, based on [5–20]

**Sheet 4.** Data sources mapped by presence of unique identifiers to assess possibility of linking.

Unique identifier	Unique data source number				
Individual ID	1	2	3	6	13
Village code	13	14			
Sub-district code	4	13			
District code	1	3	4	6	13
Health centre code	4				

Note: the unique data source number derives from Sheet 2.

Sources: Indonesia Agency for Health Research and Development, Authors, based on [5–20]

**Sheet 5.** Data sources mapped by health topics/indicators and dimensions of inequality.

Health topics	Inequality dimension																																							
	Economic status						Education						Occupation						Sex						Urban/rural						Province/region							Ethniciy		
**Reproductive health**																																								
Adolescent fertility rate	2	9					2	9					2	9					2	9					2	9					2	9								
Total fertility rate	2	9					2	9					2	9					2	9					2	9					2	9								
Contraceptive prevalence – modern methods	2	9					2	9	16				2	9					2	9	16				2	9					2	9	16							
Demand for family planning satisfied	9						9						9						9						9						9									
**Maternal health interventions**																																								
Antenatal care coverage – at least four visits	2	3	5	6	10		2	3	5	6	10		2	3	5	6	10		2	3	5	6	10		2	3	5	6	10		2	3	5	6	10	12				
Births attended by skilled health personnel	2	3	5	6	10		2	3	5	6	10		2	3	5	6	10		2	3	5	6	10		2	3	5	6	10		2	3	5	6	10	12				
Postnatal care coverage	2	3	5	10			2	3	5	10			2	3	5	10			2	3	5	10			2	3	5	10			2	3	5	10	12					
**Child health interventions**																																								
Complete basic immunization coverage	1	2	3	5	6	10	1	2	3	5	6	10	1	2	3	5	6	10	1	2	3	5	6	10	1	2	3	5	6	10	1	2	3	5	6	10	12			
Vitamin A supplementation	1	2	3	5	10		1	2	3	5	10		1	2	3	5	10		1	2	3	5	10		1	2	3	5	10		1	2	3	5	10	12				
Exclusive breasefeeding	1	2	3	5	6		1	2	3	5	6		1	2	3	5	6		1	2	3	5	6		1	2	3	5	6		1	2	3	5	6	13				
**Nutrition**																																								
Prevalence of stunting among under-5 children	1	2	3	5			1	2	3	5			1	2	3	5			1	2	3	5			1	2	3	5			1	2	3	5						
Prevalence of obesity among adults	1	2	3	5			1	2	3	5			1	2	3	5			1	2	3	5			1	2	3	5			1	2	3	5						
Prevalence of low birth weight	1	2	3	5			1	2	3	5			1	2	3	5			1	2	3	5			1	2	3	5			1	2	3	5						
**Infectious diseases**																																								
Prevalence of malaria	2	3					2	3					2	3					2	3					2	3														
Prevalence of acute respiratory infection	1	3					1	3					1	3					1	3					1	3					1	3								
Prevalence of tuberculosis (TB)																			15						15						15									
**Non-communicable diseases**																																								
Prevalence of diabetes mellitus	1	3					1	3					1	3					1	3					1	3														
Prevalence of anaemia	1	3					1	3	5				1	3	5				1	3					1	3	5													
Prevalence of hypertension	1	2	3				1	2	3				1	2	3				1	2	3				1	2	3													
**Injury**																																								
Prevalence of falls	1	3					1	3					1	3					1	3					1	3					1	3								
Prevalence of road traffic accidents	1	3					1	3					1	3					1	3					1	3					1	3								
Prevalence of serious injury							16												16												16									
**Mental health**																																								
Prevalence of population with psychosis/schizophrenia	3						3						3						3						3						3									
Prevalence of mental emotional disorder	1	3					1	3					1	3					1	3					1	3					1	3								
**Disability**																																								
Prevalence of disability	1	3					1	3					1	3					1	3					1	3					1	3								
**Child mortality**																																								
Neonatal mortality	1	10	11	13			1	10	11	13			1	10	11	13			1	10	11	13			1	10	11	13			1	10	11	13				13		
Infant mortality	1	7	10	11	13		1	7	10	11	13		1	7	10	11	13		1	7	10	11	13		1	7	10	11	13		7	10	13					13		
Under-5 mortality	1	7	10	11	13		1	7	10	11	13		1	7	10	11	13		1	7	10	11	13		1	7	10	11	13		7	10	13					13		
**Maternal mortality**																																								
Maternal mortality ratio																																								
**Healthy/unhealthy behaviour**																																								
Prevalence of current smoking	1	3	6				1	3	6	8			1	3	6	8			1	3	6	8	9	16	1	3	6	8	9		1	3	6	8	9	16				
Prevalence of alcohol consumption	1	3					1	3	8				1	3	8				1	3	8	9	16		1	3	8	9			1	3	8	9	16					
Prevalence of physical inactivity	1	3					1	3					1	3					1	3	16				1	3					1	3	16							
Prevalence of low fruit/vegetable consumption	1	3					1	3					1	3					1	3	16				1	3					1	3	16							
**Environmental health**																																								
Proportion of household using improved drinking water	1	2	3	6	13	14																			1	2	3	6	13	14	1	2	3	6	13	14				
Proportion of household using improved sanitation	1	2	3	6	13	14																			1	2	3	6	13	14	1	2	3	6	13	14				
Proportion of household using pesticide	1	2	3	6	13	14																			1	2	3	6	13	14	1	2	3	6	13	14				
**Health insurance**																																								
Proportion of population with national health insurance	3	6					3	6					3	6					3	6					3	6					3	6								
Proportion of population with province or districts health insurance	3	6					3	6					3	6					3	6					3	6					3	6								
Proportion of population with private insurance	3	6					3	6					3	6					3	6					3	6					3	6								
**Health care access**																																								
Average travel time to health centre	1	3																							1	3					1	3								
Average transportation cost to health centre	1	3																							1	3					1	3								
Inpatient utilization rate	1	3	6				1	3	6				1	3	6				1	3	6				1	3	6				1	3	6							
Outpatient utilization rate	1	3	6				1	3	6				1	3	6				1	3	6				1	3	6				1	3	6							
**Health facility**																																								
Number of hospitals by province																									4						4	12	14							
Health centre density (per 300,000 population)																									4						4	12								
Bed occupancy rate in public hospital																															12									
**Health financing**																																								
Average out of pocket health expenditure	6																								6						6									
Health expenditure per capita	6																								6						6									
**Human resource for health**																																								
Number of doctors, midwives, nurses, nutritionists, sanitarians, health promotion staff in hospitals and in health centres																									4						4	12								
Density of doctors, midwives, nurses, nutritionists, sanitarians, health promotion staff (per 10,000 population)																									4						4	12								

Source: Indonesia Agency for Health Research and Development, Authors, based on [5–20]

As countries move towards strengthening health information systems in alignment with the goal and its targets, data source mapping – a process by which data sources are systematically enlisted, their properties detailed and each source appraised for the purposes of monitoring – is an essential initial step. It has particular utility in preparing data for health inequality monitoring, but also other corollary benefits, as we explain in this short piece. We describe the elements of data source mapping, detail a recent example of the application of this process in Indonesia, and lay out the lessons of this process, in terms of both the challenges as well as the opportunities and advantages it has introduced vis-à-vis the use of data from the Indonesian health information system for equity-linked analysis.

## About the data source mapping exercise

The World Health Organization (WHO), as part of its capacity-building support to national governments and health agencies for scale-up of health inequality monitoring [3,4], developed and utilized a simple data source mapping template allowing (a) listing of data sources; (b) classification of these sources by dimension of inequality and health topic; (c) appraisal of the possibility of linkage of data from across sources; and finally (d) mapping of sources by health topic and inequality dimension (see sheets 1–5 [5–20]). This template was used for systematic data source mapping in Indonesia in 2016 as an initial step in developing a state of health inequality report for the country during the WHO-led country capacity-building workshop and the subsequent period [21]. Led by the Indonesia Agency for Health Research and Development (IAHRD) and in consultation with other stakeholders, the data source mapping template was filled out, drawing from all relevant data sources.

On the first sheet, 11 population-based surveys were identified, including RISKESDAS, the Basic Health Research Survey, which has its basis in an earlier household-based survey that has been in place since 1986. In addition, topic-based surveys on tuberculosis prevalence (2004, 2014), and the WHO Global Adult Survey (GATS; 2011) and Youth Tobacco Surveys (GYTS; 2006, 2009, 2014) were included. From beyond the health sector, SUSENAS, the National Socioeconomic Survey (started in 1979 and administered annually since 1989) and the Village Potential Survey (PODES), administered roughly triennially since 1983, were listed. The decennial census was also listed, which has been implemented since 1971. Indonesia’s vital registration and sample registration system were also included.

Of these sources, 16 were enlisted in the second sheet with dimensions of inequality. Province/region was the most commonly available disaggregation across data sources (available in 15 out of 16), closely followed by sex and urban/rural place of residence. The least commonly available dimension of inequality was race/ethnicity, indicated in the population census. Overall, it appeared that RISKESDAS seemed to have a fairly high number of dimensions of inequality, alongside SUSENAS, and followed by SIRKESNAS.

In the third sheet, 18 health topics ranging from reproductive health to non-communicable diseases, and maternal mortality to health financing were enlisted, with 50 indicators falling under these topics. For each indicator and topic, unique data sources were mapped. It was noted that the most commonly available health topics across data sources were environmental health, maternal health interventions, child health interventions, nutrition, and child mortality. In contrast, the fewest data sources were seen for injury and mental health, disability, and health financing. From this mapping, it could immediately be seen that RISKESDAS had the greatest potential to serve as the basis for reporting on health inequality, given that across multiple rounds, a wide range of health topics was covered and the availability of dimensions of inequality was also reported.

The next sheet assessed unique identifiers, which are codes for individuals or geographical units (like district or province) that may be used in multiple data sources. These identifiers can then be used to link datasets; e.g. unique identifiers at the district level may be used to link health facility access from a survey to district development rankings using the census. Indonesia has unique identifiers for individuals, as well as for village, sub-district, district and province across multiple data sets. By far, the most commonly available unique identifiers were at the individual and district level, seen in five different data sources each. The census had the largest number of unique identifiers (by individual ID, village code, sub-district code and district code).

The consolidated mapping confirmed what was already becoming evident: RISKESDAS (2007, 2010, 2013) seemed to have the greatest potential to be used as the first and main data source for health inequality analysis and reporting. This matrix allowed more specific reflection on what topics and inequality dimensions made the most sense to use for the inequality report. The report *State of health inequality*: *Indonesia* consisted of 11 topics, namely the dimensions of inequality wereused in the final report; namely, economic statusPublic Health Development Index (PHDI); reproductive health; maternal, newborn and child health; childhood immunization; childhood malnutrition; child mortality; infectious diseases; environmental health; non-communicable diseases (NCDs), mental health and behavioural risk factors; and disability and injury; as well as health facility and personnel. Eight dimensions of inequality were used in thereport; namely, economic status, education, occupation, employment status, place of residence, sex, age and subnational region [21].

## Lessons learned

The data source mapping exercise not only allowed a critical review and appraisal of the range and granularity of data sources in the country of Indonesia, but also helped guide the selection of these data sources for health inequality data preparation, analysis, reporting, and the setting up of a monitoring framework. This process has been useful from the perspective of embedding an equity lens in both appraising and designing health information systems at the national level and offers potential for replication/scaling at lower administrative levels (i.e. the district, etc.).

Nevertheless, the process was not without challenges. First of all, data source mapping cannot directly address challenges of data quality – a matter that, as in all research, has to be ensured during data collection and management for each source. If there are strong reservations about the quality of data from a particular source, this could be noted during data source mapping and built into considerations at the stage of data extraction and analysis. The more we come to rely on certain data sources, the greater our impetus to ensure that it is of high quality over time. Further, it was crucial to note that just because a data source is mapped, this does not mean that access to one or more data sources will be obtained [22]. Another challenge was that surveys themselves have changed from year to year, based on evolving governmental priorities. It therefore could not be assumed that the same health topics were covered from year to year and, even if they were, they may have been operationalized using slightly different indicator definitions or referent time periods. For example, SUSENAS had a slightly different definition of indicators for birth attendance and immunization as compared to RISKESDAS and therefore those two data sources were not fully comparable. Similarly, the same dimension of inequality (e.g. economic status) could be operationalized in very different ways across data sources, or even across years in the same data source (in some cases this reflected policy shifts and priorities; for instance, when district or provincial boundaries changed). For example, economic status subgroups in RISKESDAS 2010 were derived from household expenditure, yet in RISKESDAS 2013 the subgroups were determined based on a 12-item scale of household assets. Finally, even if separate data sources have topics and dimensions of inequality, the ideal scenario would be one where they may be linked with a unique identifier at the lowest possible level (i.e. individual) so that individual-level inferences can be made. Administratively, however, data sources may not have unique identifiers or the same unique identifier across data sources. This has to be catered to in the data source mapping exercise and, of course, affects the interpretation and application of the matrix created in the exercise.

Notwithstanding these challenges, data source mapping allowed careful inspection and appraisal of data sources, identification of gaps, reflexivity about how they are linked, what they covered and their degree of meaningfulness from an inequality monitoring perspective, and, perhaps more broadly, in the design of equity-centric policy. Here, the difference between inequity and inequality bears mentioning: health inequities are systematic health differences between different population subgroups that are unjust, unfair and avoidable, while health inequalities are observed health differences between population subgroups [4]. Monitoring health inequalities can track how changes in health in the whole population are realized by different population subgroups. It can help identify the state of health and access to health services in the disadvantaged population subgroups and inform policy responses that are equity-oriented. Data source mapping may also be a starting point for identifying where data is lacking and indicators do not exist. Indeed, any comprehensive equity-oriented policymaking should avoid basing itself solely on data that is easily available, as very often the data most relevant to equity is the hardest to quantify and/or gather.

To conclude, data source mapping can inaugurate a broader conversation about what more needs to be done to enhance the range and depth of topics in health that are currently covered by various information systems, how granular the data on these topics are and are not, and what procedures have to be in place to link data sources. Such a process is fairly customizable and open ended – allowing health inequality assessment in Indonesia or in other countries – and thus offers promise for application and replication. The task of addressing health inequity, of course, encompasses much more than this; it requires careful appraisal and tailored application of policy and programme interventions designed to reduce inequities. Data source mapping, which reveals the availability of data required for monitoring, is a necessary but insufficient step in addressing inequities at various levels of decision-making. Such a process must necessarily be nested within a broader analytical framework for equity-oriented decision-making informed by evidence, with political commitment and public participation to truly follow the letter and spirit of the Sustainable Development Goals.

## Paper context

Data source mapping – a process whereby data sources are systematically enlisted, their properties detailed in relation to health indicators and inequality dimensions, and each source appraised for the purposes of monitoring – is an essential initial step for health inequality monitoring. We outline a simple process along with a template for data source mapping and evaluate its application in Indonesia. This is one key component in a larger process by which data may be used for equity-oriented monitoring and policymaking at the national level.
